# Very late recurrence of Diethylstilbestrol - related clear cell carcinoma of the cervix: case report

**DOI:** 10.1186/s40661-015-0010-5

**Published:** 2015-07-17

**Authors:** Ablavi Adani-Ifè, Emma Goldschmidt, Pasquale Innominato, Ayhan Ulusakarya, Hassan Errihani, Philippe Bertheau, Jean François Morère

**Affiliations:** Department of Oncology, National Institute of Oncology, Avenue Allal El Fassi, BP 6542, Rabat, 10100 Maroc; Department of Oncology, Paul Brousse University Hospital AP-HP, 12-14 Avenue Paul Vaillant Couturier, 94800 Villejuif, France; Laboratory of Cytopathology, Saint Louis Hospital AP-HP, Avenue Claude Vellefaux, 75010 Paris, France

**Keywords:** Clear cell adenocarcinoma, Cervix, Diethylstilbestrol, Recurrence

## Abstract

Clear cell adenocarcinoma of the cervix is a rare tumor of the lower genital tract. It has been described in young women with a history of intra uterine exposure to diethylstilbestrol. This tumor is characterized by a greater tendency for late recurrences. In this article, we report the case of one exposed-patient who developed recurrence as liver metastases, 24 years after the initial treatment. This case demonstrates the need and the importance for continued follow-up in individuals prenatally exposed to diethylstilbestrol.

## Background

Clear cell adenocarcinoma of the cervix is an uncommon malignancy accounting for 4–9 % of cervical adenocarcinomas which represent about 5–10 % of all tumors of the cervix [1, 2]. It has been first described in young women exposed *in utero* to Diethylstilbestrol (DES) by Herbst et al. [3] and its incidence is in on the order of 1.0 per 1000 exposed persons [4, 5]. Clear cell adenocarcinomas of the lower genital tract have a greater tendency to recur late and develop metastases in distant sites more frequently than squamous cell carcinomas [6–8]. We report here the case of one patient who developed recurrence 24 years after the initial treatment.

## Case presentation

In July 1990, a 20-year-old woman presented with abnormal vaginal bleeding. Her past medical history was only significant for *in utero* DES exposure. Physical examination revealed a budding tumor involving the anterior lip of the uterine cervix. The pelvic Computed Tomography (CT) scan showed an enlargement of the cervix which was deviated to the right. The mass came in contact with the rectal wall but did not invade the bladder or the parameters. Biopsy confirmed the diagnosis of clear cell adenocarcinoma of the cervix. Disease extention evaluation including chest and abdominal scan was normal. A laparotomy with bilateral ovarian transposition and iliac lymphadenectomy was performed. The pathological examination of resected lymph nodes was negative. After the initial surgery, the patient was treated with brachytherapy (65 Grays). Because of an insufficient tumor response to the brachytherapy, the patient underwent two cervical conizations. After the second conization, surgical margins were negative for malignancy. The patient was then followed up regularly with colposcopic evaluation and annual Pap smear.

**Fig. 1:**
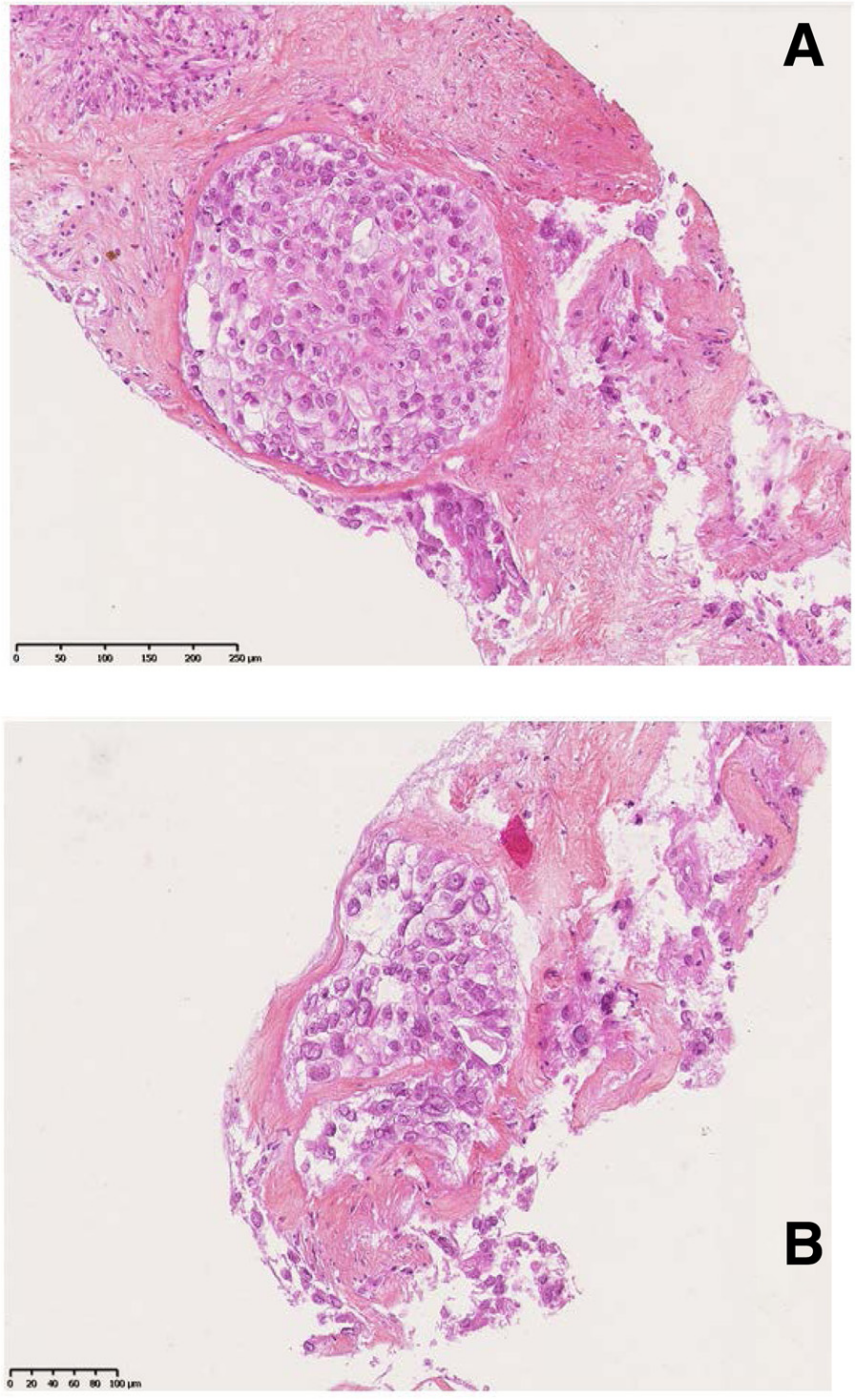
Histological findings. Biopsy of a liver metastasis showing glandular tumor mass composed of tubular structures with large cells and clear cytoplasm (Hematoxylin and eosin staining (**a**) and (**b**) with high magnification)

**Fig. 2:**
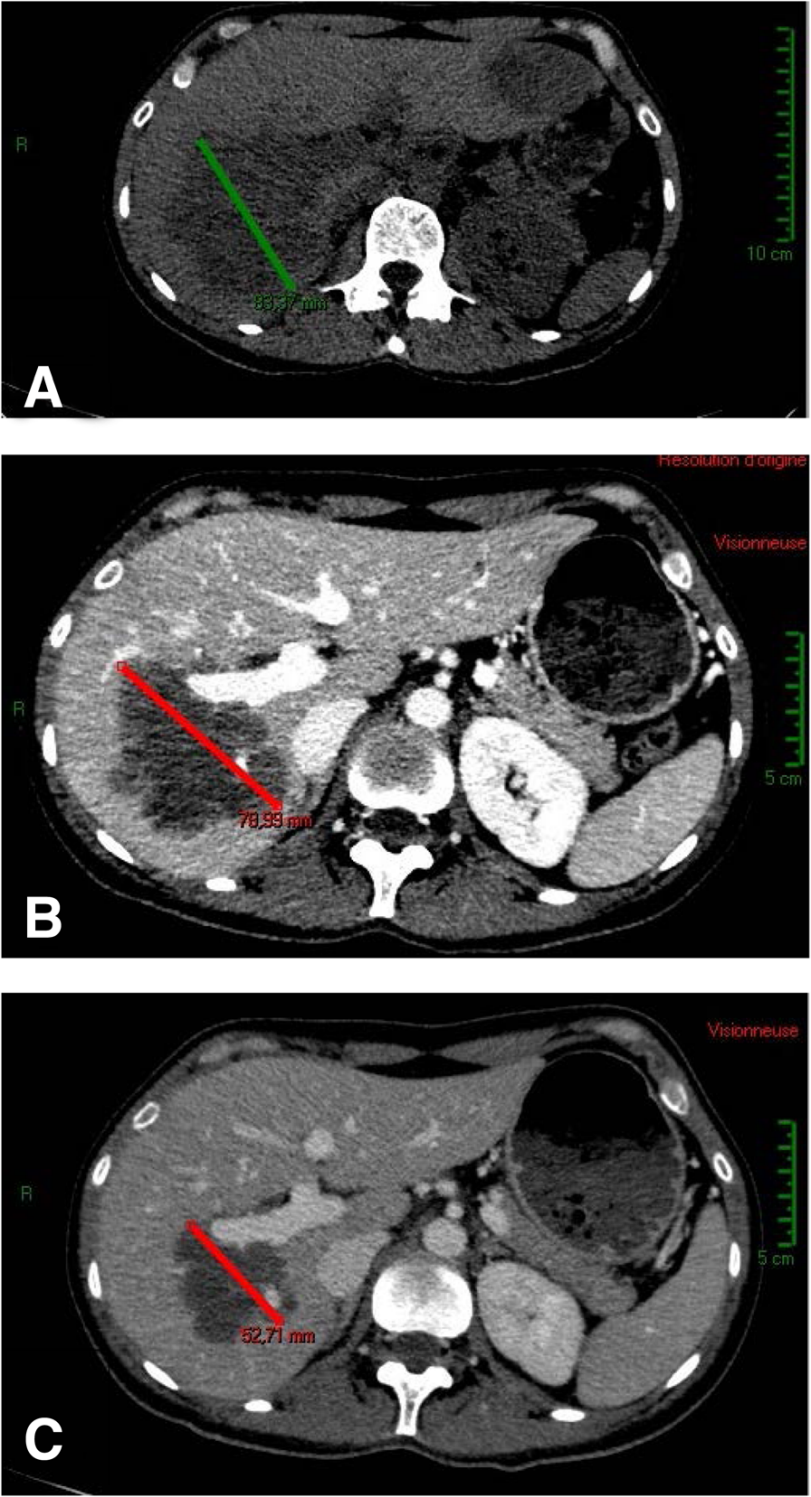
Before and after the treatment. CT scans showing hepatic metastases before the treatment (**a**), and the tumor size reduction after 3 cycles of chemotherapy (**b**) and after 6 cycles (**c**)

Despite the conservative treatment, the patient was unable to conceive. Fourteen years later in December 2004, she complained of metrorrhagia. Pelvic ultrasound revealed an abnormal uterine mass of 28 mm. The patient underwent radical hysterectomy and right annexectomy. The pathological analysis of the mass revealed a leiomyoma and the right annex was normal. The patient was followed up regularly and was considered to be free of disease until February 2014 when she developed abdominal pain and weight loss. Abdominal CT scan showed multiple hepatic masses and peritoneal carcinomatosis. A hepatic percutaneous biopsy revealed a tumor with tubular pattern consisting of large and polygonal cells with clear or eosinophilic cytoplasm CK7+, CK20-, TTF1-, ER-, PR-, HER2-, CDX2- and CK5/6- (Fig. 1). Based on these characteristics, the tumor was considered as metastatic diffusion of the previously treated clear cell adenocarcinoma. Imaging studies for the relapsed disease including a FDG-PET-scan confirmed hepatic and peritoneal lesions. The patient received combination chemotherapy regimen including weekly paclitaxel (80 mg/m^2^), carboplatin (AUC = 5) and bevacizumab (7.5 mg/kg) both administered every three weeks. To date, she has received 7 cycles of this combination with excellent tolerance and the follow up CT scans have showed a partial response with reduction of tumor size after 3 and 6 cycles (Fig. 2).

## Discussion

Diethylstilbestrol (DES) is an oral synthetic nonsteroidal estrogen that was used to prevent miscarriage, premature birth and other pregnancy complications between 1938 and 1971 in the United States [9] and until early 1980’s in various European countries [9, 10]. Exposure to DES during a critical period of organogenesis disturbs the developing uterine muscle layers, causes abnormalities of the uterotubal junction, and prevents stratification of the vaginal epithelium and resorption of vaginal glands, resulting in vaginal adenosis [10]. In female offspring, *in utero* exposure to DES has been associated with potential risks of cervicovaginal clear cell adenocarcinoma, congenital anomalies and epithelial changes of the reproductive tract, subfertility and adverse pregnancy outcomes, earlier age at menopause, breast cancer and cervical intra epithelial neoplasia [10].

DES related clear cell adenocarcinoma usually occurs between the age of 15 and 27 with a median of 19 years [4] and have a predilection for the ectocervix and upper third of the vagina [11]. Pathologically, the tumors can display solid, tubular, cystic and papillary patterns or mixed patterns [12, 13] with the tubular-cystic pattern being the most common presentation [13]. Survival rates approaching 90 % can be expected in patients with clear cell adenocarcinoma with papillary and tubulocystic features while tumors with more solid pattern have less favorable prognosis [14]. Most cases of clear cell carcinoma related to DES exposure have been diagnosed at stage one or two [5]. Patients with early stage are highly curable with surgery or radiotherapy or combination of both modalities [15–17]. However, clear cell adenocarcinoma can also be diagnosed in unexposed women. It can occur in older women [12, 18] and in about 25% of cases in young women, there is no history of maternal medication [19].

Most recurrences of clear cell adenocarcinoma of the vagina and cervix are diagnosed within the 3 years after primary tumor treatment [8] but late recurrences have been reported with few cases 8 years after initial diagnosis [7, 14, 20, 21]. To date the latest recurrence reported in DES exposed patients is 19 years after initial therapy [11]. Here, we present the case of a woman with intra uterine DES exposure who developed recurrence as distant metastases without local relapse, 24 years after initial curative treatment. In 1991, Goodman and coll reported a local recurrence of clear cell adenocarcinoma of the vaginal remnant presenting 20 years after initial surgery. Basing on the 20 years disease -free period, the absence of nodal, lymphatic or vascular involvements and the absence of distant spread in their case, they suggested a new primary tumor rather than a late recurrence [22]. In our patient, the absence of local relapse suggests the possibility that quiescent tumor cell may have been initially present in the liver and peritoneum and became activated after a prolonged interval and/or evolved slowly and finally became symptomatic.

In the cases reported by Herbst et al. [8], recurrences were more frequent in the pelvis (60 %), the lungs (36 %) and supraclavicular lymph nodes (12 %). One patient was reported with cerebellar metastases [20] but liver metastases from DES related clear cell adenocarcinoma of cervix have never been described. Because of a similar histological morphology, this diagnosis can be confused with that of primary clear cell carcinoma of liver, which is a particular and rare histological type of hepatocellular carcinoma [23, 24]. However, most of primary clear cell carcinoma of the liver occur in patients with liver cirrhosis [25] and the cases reported in patients with normal liver are uncommon [26]. Nevertheless, for the present case, immunostaining of biopsy samples excluded primary clear cell carcinoma of liver.

Local recurrences of clear cell adenocarcinoma of the cervix can be effectively treated with surgery, radiotherapy or combined modality [21]. Surgery or radiation therapy can be used to treat also limited metastatic recurrence. In disseminated recurrent disease, systemic chemotherapy including various cytotoxic drugs (alkylant agents, 5-fluorouracil, adriamycine, vinca-alcaloids, actinomycine D, cisplatin) or progestational agents has been used [8, 21] but no effective regimen is currently considered as the reference treatment [8, 21]. However paclitaxel has been administrated to one patient, and has permitted to obtain stable disease on CT scan and the decrease of the initially high CA 125 tumor marker [11]. Our patient is being treated with a combination of paclitaxel, carboplatin and bevacizumab which is an anti-angiogenic (anti Vascular Endothelial Growth Factor) monoclonal antibody. This regimen is active, inducing a clinical improvement (symptom disappearance) and a morphologic partial response.

## Conclusions

To summarize, this case represents the longest reported disease-free interval till recurrence and the first description of metastatic liver disease of DES related clear cell adenocarcinoma of the cervix. It reemphasizes the necessity of long term surveillance of DES exposed women and confirms previous reports recommending the importance of frequent follow-up examination not only of the pelvis but also of all distant potential sites of metastasis. It also shows that treatment with paclitaxel, carboplatin and bevacizumab can be an effective and safe therapeutic option for treating recurrence of this rare tumor.

## Consent

Written informed consent was obtained from the patient for publication of this Case report and any accompanying images. A copy of the written consent is available for review by the Editor-in-Chief of this journal.
